# Internet of Things (IoT) technology and its application in Ophthalmology

**DOI:** 10.22336/rjo.2024.01

**Published:** 2024

**Authors:** Consuela-Mădălina Gheorghe

**Affiliations:** * Philologist, Certified translator

According to Oracle, the famous multinational American computer company, the network of physical items, or “things”, that are implanted with sensors, software, and other technologies to communicate and exchange data with other devices and systems over the internet, is known as the Internet of Things (IoT) (https://www.oracle.com/internet-of-things/what-is-iot/). These gadgets might be anything from simple domestic items to highly advanced industrial instruments. Experts predict that the current number of linked IoT devices will increase to 22 billion by 2025.

IoT is important because, nowadays, every object from our surroundings can be connected to the internet, thus communication between people, things, and processes is also enhanced.

In healthcare, IoT asset monitoring offers many benefits for all the members of the hospital as they need to know the exact location of the patient-assistance assets, which are equipped with IoT sensors, making it easy for any patient to find the nearest available asset. With the help of these IoT sensors, the assets can be tracked to verify their proper usage, prevent theft, and keep track of preventive maintenance and housekeeping. Real-time asset-tracking IoT solutions can improve both patient experience and workflow in many fields of medicine and also in ophthalmology (https://www.hospitalinformationsystem.com/iot-solutions-eye-hospitals/).

zPatient workflow can be ensured by using Bluetooth Low Energy (BLE) devices. Among the devices that use BLE are thermometers, heart rate monitors, blood pressure monitors, etc., and in ophthalmology, optical handheld magnifiers, used by visually impaired adults, the technology facilitating infrequent short-range wireless data communication between devices, powered by a dime-sized battery.

The demand for IoT in ophthalmology services has increased during the COVID-19 pandemic, when, to reach the patients and be able to treat them, ophthalmologists appealed to remote and digital means, these smart technologies proving very beneficial by providing better treatments although the ophthalmologist and the patient were not in the same room or place anymore (without physical intervention). Moreover, IoT technology has proved to be profitable by reducing the monetary (the cost per se) and nonmonetary costs (physical, and psychological costs) of the patients and being available anytime and anywhere.

IoT technology is currently helping many healthcare organizations to meet their objectives, thus, two branches have appeared: the Internet of Healthcare Things (IoHT) and the Internet of Medical Things (IoMT). IoHT offers the possibility of combining patient data in the ophthalmology department in hospitals or clinics to improve efficiency, optimize resources, and minimize patient ophthalmological health deterioration. Internet of Medical Things (IoMT) is a next-generation bio-analytical tool that combines network-linked biomedical devices with a software application for advancing human health (Manickam P, Mariappan SA, Murugesan SM, Hansda S, Kaushik A, Shinde R, Thipperudraswamy SP. Artificial Intelligence (AI) and Internet of Medical Things (IoMT) Assisted Biomedical Systems for Intelligent Healthcare. Biosensors (Basel). 2022 Jul 25;12(8):562. doi: 10.3390/bios12080562. PMID: 35892459; PMCID: PMC9330886). By using smart tools, medical problems can be diminished or eradicated for many diseases, including ophthalmological diseases (Al-Kahtani MS, Khan F, Taekeun W. Application of Internet of Things and Sensors in Healthcare. Sensors (Basel). 2022 Jul 31;22(15):5738. doi: 10.3390/s22155738. PMID: 35957294; PMCID: PMC9371210).

In conclusion, it can be stated that IoT is a growing area of research in healthcare, and, in Ophthalmology, IoT solutions can be used for the early detection and prevention of eye diseases, increase efficiency in eye clinics and hospitals, enhance the accessibility of eye care, etc. 


**Assist. Prof. Consuela-Mădălina Gheorghe, PhD, Philologist, Certified translator**


